# Trigeminal neuralgia-like pain in a vagus nerve stimulation *super-responder* with drug-resistant idiopathic generalized epilepsy: A case report

**DOI:** 10.1016/j.ebr.2025.100844

**Published:** 2025-12-24

**Authors:** Javier Peña-Ceballos, Tenzin Choekyi, Aoife Walsh, Breege Staunton-Grufferty, Vicente Casitas-Hernando, Donncha O’Brien, Norman Delanty

**Affiliations:** a Department of Neurology, Beaumont Hospital, Dublin, Ireland; b Department of Neurosurgery, Beaumont Hospital, Dublin, Ireland; c FutureNeuro Research Ireland Centre, Dublin, Ireland; d School of Pharmacy and Biomolecular Sciences, Royal College of Surgeons in Ireland, Dublin, Ireland

**Keywords:** Idiopathic generalized epilepsy, Drug-resistance, Vagus nerve stimulation

## Abstract

We report a 38-year-old man with drug-resistant idiopathic generalized epilepsy (IGE) who became seizure-free after being implanted with vagus nerve stimulation (VNS). He developed a trigeminal neuralgia-like pain (TNLP) time-locked with ON stimulations. TNLP did not cease after restoring the initial stimulation parameters. After an unsuccessful trial with carbamazepine, the patient reported a moderate improvement with pregabalin. He remained seizure-free, being able to partially rationalize his ASM therapy and the ability to start driving lessons, with the normalization of his routine electroencephalogram (EEG).

## Highlights

• VNS is an effective adjunctive treatment in drug-resistant IGE.
• TNLP is a rare side effect of VNS therapy.
• Pharmacological treatment may be needed if TNLP persists after reprogramming.

## 1 Introduction

Vagus nerve stimulation (VNS) therapy is a safe and effective non-pharmacological option for patients with drug-resistant epilepsy (DRE) with increased tolerability and efficacy over time [1]. Trigeminal neuralgia-like pain (TNLP) is a rarely reported side effect of VNS therapy [2,3,4,5]. VNS therapy has been predominantly used in patients with focal epilepsy. However, given the growing evidence, VNS is increasingly used also in drug-resistant generalized epilepsies [6]. Whereas VNS therapy has been traditionally considered a palliative technique, seizure freedom rates of 8–11 % have been reported in recent studies [7,8]. Factors predicting seizure freedom include generalized seizures and epilepsy onset after the age of 12 [8,9].

## 2 Case presentation

**Fig. 1:**
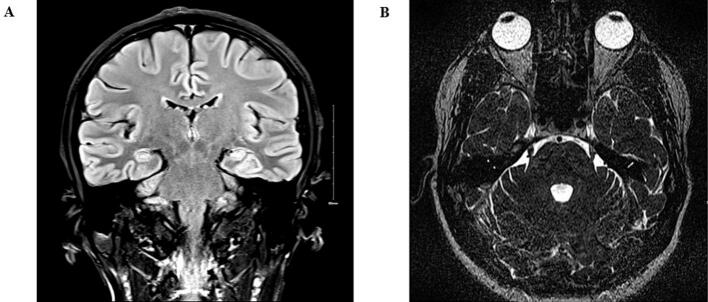
Examples of 3 T brain magnetic resonance imaging (MRI). A: T2 Coronal flair without an evident structural abnormality. B: T2-weighted axial did not reveal vascular compression of the trigeminal nerve.

**Fig. 2:**
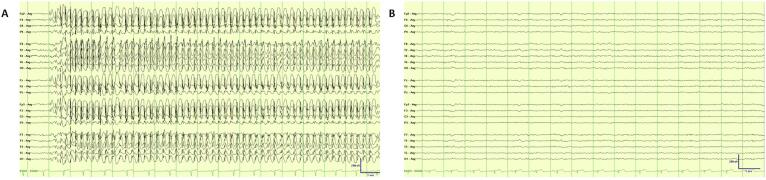
Example of an ictal and routine electroencephalography (EEG). A. Example of a typical absence seizures during admission into the video-electroencephalography (video-EEG). Natus Neuroworks® EMU40EX™. Montage: Average. Low-frequency filter: 1 Hz, High-frequency filter: 70 Hz, Notch: 50 Hz, Sensitivity: 10 μV/mm, Timebase: 30 mm/sec. B. A normal routine EEG after vagus nerve stimulation (VNS) implantation. Natus Neuroworks® EMU40EX™. Montage: Average. Low-frequency filter: 1 Hz, High-frequency filter: 70 Hz, Notch: 50 Hz, Sensitivity: 10 μV/mm, Timebase: 30 mm/sec.

We present a 38-year-old right-handed man with a diagnosis of drug-resistant idiopathic generalized epilepsy (IGE) who developed a delayed onset of TNLP *time-locked* with ON time stimulations of VNS therapy. He initially presented to our epilepsy clinic after his first lifetime convulsion at the age of 24. During the first consultation, he reported brief daily lapses in conversation over the preceding months. He did not have early risk factors for epilepsy. He had no significant medical history, and his neurological examination was unremarkable. His initial epilepsy-protocol brain magnetic resonance imaging (MRI) did not reveal any structural abnormality (Fig. 1A). An early-morning electroencephalogram (EEG) demonstrated a burst of 3–4 Hz generalized spike and wave discharge (GSWD) of up to 6 s, consistent with a diagnosis of late-onset IGE. Initial trials of antiseizure medication (ASM) with lamotrigine, levetiracetam, and then clobazam were adequately tolerated but did not lead to a sustained reduction in daily absence seizures. Serial early-morning routine EEGs revealed frequent interictal GSWD. Given the persistence of daily absence seizures despite polytherapy with brivaracetam, ethosuximide, sodium valproate, and zonisamide, the patient was referred to our video-EEG monitoring unit to quantify seizure frequency and exclude combined generalized and focal epilepsy. During the admission, several absence seizures of up to 22 s were captured, characterized clinically by brief interruption of activity, staring, and subtle eyelid blinking (Fig. 2A).

He was implanted with a VNS SenTiva™ Model 1000 device without perioperative complications. Two weeks later, his VNS therapy was switched on, and an automatic titration plan was initiated, with changes in the output current (OC) of 0.25 mA every 2 weeks until reaching a target of 1 mA. Other stimulation parameters include a stimulation frequency (SF) of 20 Hz, a pulse width (PW) of 250 microseconds (μs), a time ON of 30 s, and a time OFF of 5 min (duty cycle 10 %). The AutoStim was programmed at the same output current, SF of 20 Hz, PW of 250 μs, time ON of 30 s, and with a threshold of 40 %. The magnet mode was set to an OC 0.25 mA higher at each adjustment step, PW of 500 μs, and time ON of 60 s. At the 12-week review, he reported being seizure-free since VNS was commenced, but complained about long-standing *“brain fog”*. During the 12-week review, the OC was first increased to 1.25 mA and was well tolerated. In order to achieve therapeutic settings and given this favorable initial tolerance, the OC was subsequently increased to 1.5 mA during the same consultation. We increased the OC of the AutoStim and magnet to 1.5 mA and 1.625 mA, respectively. However, he stated that he had not used the magnet yet. We also started weaning the zonisamide to zero.

Two weeks later, he contacted us due to an excruciating pain affecting the left-sided mandibular area, *time-locked* with the ON time stimulations. Dental examination was inconclusive. At the 24-week post-implantation follow-up, we reduced the OC to 1 mA, but no clinical improvement occurred in the following weeks. In addition, during the 24-week review, the AutoStim-related stimulations accounted for < 1 % of stimulations, and the OC was also reduced to 1 mA. At the 35-week review, we further decreased the PW to 130 μs, yet his symptoms remained unchanged. MRI of the trigeminal nerve ruled out vascular compression (Fig. 2B). Interestingly, during the MRI with the VNS switched off, the patient reported no episodes of paroxysmal pain. TNLP did not cease after a trial of carbamazepine, but improved moderately with pregabalin. His seizure control did not deteriorate after trials of carbamazepine or pregabalin. At the 54-week post-implantation review, the patient has remained seizure-free, and for the first time since seizure onset, his early-morning EEG did not show any interictal abnormality (Fig. 1B). His partner did not notice lapses in conversation as she had previously, and he has commenced driving lessons. Table 1 summarizes the VNS parameters management and the ASM regimen at different weeks post-implantation. Considering that he did not use the magnet and the AutoStim results accounted for <1 % of daily stimulations, we did not include these parameters in Table 1.

**Table 1:** Summary of stimulation parameters, clinical response, adverse events, and antiseizure medication regimen at different follow-ups post-implantation.

Weeks post-implantation	VNS Stimulation parameters						Overall seizure frequency response	Adverse effects	Manual/automatic programming	Antiseizure medication/24 h
	Output current (mA)	Frequency (Hz)	Pulse width (μs)	Time ON (s)	Time OFF (min)	Duty cycle (%)				
2	0.25	20	250	30	5	10 %	Not applicable	None	Automatic	BRV 300 mg ETX 1000 mg VPA 1800 mg ZNS 600 mg
12	1	20	250	30	5	10 %	Seizure-free	None	Automatic	BRV 300 mg ETX 1000 mg VPA 1800 mg ZNS 600 mg
14	1.5	20	250	30	5	10 %	Seizure-free	TNLP	Manual	BRV 300 mg ETX 1000 mg VPA 1800 mg ZNS 500 mg
24	1	20	250	30	5	10 %	Seizure-free	TNLP	Manual	BRV 300 mg ETX 1000 mg VPA 1800 mg ZNS 100 mg
35	1	20	130	30	5	10 %	Seizure-free	TNLP	Manual	BRV 300 mg ETX 1000 mg VPA 1800 mg
39	1	20	130	30	5	10 %	Seizure-free	TNLP	Manual	BRV 300 mg ETX 1000 mg VPA 1800 mg
54	1	20	130	30	5	10 %	Seizure-free	TNLP *No improvement after trial of CBZ	Manual	BRV 300 mg ETX 1000 mg VPA 1800 mg CBZ 400 mg for TNLP
65	1	20	130	30	5	10 %	Seizure-free	TNLP *Moderate improve after trial of PGB	Manual	BRV 300 mg ETX 1000 mg VPA 1800 mg PGB 100 mg for TNLP

Abbreviations: BRV = Brivaracetam, CBZ= Carbamazepine, ETX = Ethosuximide, Hz= Hertz, mA = Milliamp, PGB= Pregabalin, TNLP = Trigeminal neuralgia-like pain, VNS = Vagus nerve stimulation, VPA= Sodium valproate, μs = Microseconds.

## 3 Discussion

Our case report underscores that VNS might be an effective treatment in patients with drug-resistant IGE, including those with absence seizures as the main seizure type [10]. There is growing evidence supporting the use of neurostimulation in generalized epilepsies, given its potential modulatory effect on thalamocortical networks involved in the generation of GSWD [6,11]. The antiseizure mechanism of VNS is not fully understood. Positron emission tomography studies show that VNS leads to changes in cerebral blood flow that facilitate synaptic activity across several regions, including the thalamus and its thalamocortical projections to the cortex [12]. In functional MRI studies, VNS enhanced thalamic activation and thalamocortical connectivity [13,14].

We consider this patient a *super-responder*, not only because he achieved seizure freedom, but also because of partial rationalization of ASM therapy, normalization of the EEG, and the ability to start driving lessons [15]. His ASM therapy was partially rationalized, as before VNS implantation, he was treated with brivaracetam, ethosuximide, sodium valproate, and zonisamide. Around week 24 post-implantation, zonisamide was completely discontinued. Pregabalin, which is not commonly used for absence seizures, was introduced for pain management at week 65 post-implantation, following 45 weeks of seizure freedom. Accordingly, we do not attribute any effect on seizure control to pregabalin.

Our patient achieved seizure freedom at an OC lower than the suggested optimal settings [16,17]. A recently published study found that *super-responders* achieved seizure freedom at significantly lower stimulation settings [18]. *Super-responders* had a significantly shorter epilepsy duration [18], aligning with previous studies that associated earlier VNS implantation with better seizure and non-seizure outcomes [9,19,20,21,22]. Interestingly, Tamura and colleagues suggested potential intrinsic features that might predispose a positive response to VNS therapy [18]. A recent study found that baseline thalamic resting-state functional connectivity was associated with long-term seizure frequency reduction in patients with focal epilepsy, supporting the growing evidence of potential intrinsic biomarkers for treatment response [23].

TNLP is a rare side effect that can occur up to several months after the last changes in stimulation settings and usually improves once the settings are reversed [2,3,4,5]. The potential mechanism of TNLP remains unknown. TNLP might be explained by the intermittent activation of trigeminal sensory pathways via vagal projections towards the spinal trigeminal nucleus [3,4]. In a literature review of 7 patients, TNLP occurred with OC at 0.5–2 mA from days to up to 4 months after the latest VNS changes [2]. Deactivation of the VNS should be avoided in patients with a positive response given the risk of seizure relapse [2,5]. In our patient, TNLP emerged 2 weeks after titrating the OC from 1 to 1.5 mA during the same consultation, which remains a possible trigger. The later reduction of OC and PW did not reverse the symptoms, as previously reported in other series [2,3,4]. A prompt clinical review is advised after the onset of TNLP to restore the initial stimulation settings and evaluate whether symptoms cease. If TNLP persists, we recommend referring patients for a trigeminal MRI and dental examination to rule out other causes. Given the rarity of this side effect, it remains unclear whether it is attributable to our dosing management or to the 10-week delay to restore the initial stimulation parameters. In our clinical practice, we have previously increased the OC dosing by 0.5 mA during the same consultation if the initial increment of 0.25 mA was well tolerated in patients who had previously reached a minimum OC of 1 mA, without the emergence of TNLP. Nonetheless, we advise adherence to guidelines that recommend increasing the dose by 0.25 mA every 14 days or by 0.125 mA every 7 days. In other case reports, VNS was deactivated to relieve symptoms for a later rechallenge; however, this led to seizure relapse [2,5]. In addition, we recommend exploring patient preferences, rapid restoration of initial stimulation parameters and considering pharmacological pain management if reducing OC is ineffective, instead of decreasing the PW, since either reducing the OC or PW may impact seizure control [24]. Subsequently, reducing the PW to 130 microseconds will require an increase in the OC, which can worsen symptoms [24]. The first-line pharmacological pain management of trigeminal neuralgia with carbamazepine and oxcarbazepine should be avoided in patients with IGE, given the risk of seizure exacerbation [25].

Overall, this case highlights the effectiveness of VNS in drug-resistant IGE and underscores the importance of recognizing and managing rare stimulation-related side effects such as TNLP. We recommended following the dosing guidelines and considering pharmacological pain management if TNLP persists after adjusting the stimulation parameters.

## Consent

We confirm that the patient has given his consent for publication.

## Declaration of generative AI in scientific writing

The authors have not used any type of generative AI during the preparation of this manuscript.

## Ethical statement

Ethical review was waived because of the nature of the case report, and informed consent was obtained from the patient.

## CRediT authorship contribution statement

**Javier Peña-Ceballos:** Writing – original draft, Visualization, Methodology, Investigation, Formal analysis, Data curation, Conceptualization. **Tenzin Choekyi:** Writing – review & editing, Data curation. **Aoife Walsh:** Writing – review & editing, Data curation. **Breege Staunton-Grufferty:** Writing – review & editing. **Vicente Casitas-Hernando:** Writing – review & editing. **Donncha O’Brien:** Writing – review & editing. **Norman Delanty:** Writing – review & editing, Visualization, Validation, Supervision, Project administration, Methodology, Funding acquisition, Conceptualization.

## Funding

This study did not receive any specific grant from funding agencies in the public, commercial, or not-for-profit sectors.

## Declaration of competing interest

The authors declare the following financial interests/personal relationships which may be considered as potential competing interests: JPC served as a speaker and received travel expenses from Angelini Pharma, Jazz Pharmaceuticals and LivaNova PLC. TC received travel expenses from Jazz Pharmaceuticals and Angelini Pharma. Over the last three years, ND has served as a paid advisor and/or speaker for Actio Biosciences, Angelini Pharma, Eisai, LivaNova PLC, UNEEG Medical, and Jazz Pharmaceuticals. The rest of the authors reported no conflict of interests for this article.
